# Unexpected Rhythm: Supraventricular Tachycardia Unveiled in a Neonate Diagnosed at Delivery

**DOI:** 10.7759/cureus.65710

**Published:** 2024-07-29

**Authors:** Balakrushna Garud, Gaurav Kumar, Sanjay Chavan, Shradha Salunkhe, Ganta Chandrashekhar Reddy

**Affiliations:** 1 Pediatrics, Dr. D. Y. Patil Medical College, Hospital & Research Centre, Dr. D. Y. Patil Vidyapeeth (Deemed to be University), Pune, IND

**Keywords:** fetal heart rate, neonate, maternal-child health, calcium channel blockers or adenosine and supraventricular tachycardia, supraventricular tachycardia

## Abstract

Neonatal supraventricular tachycardia (SVT) poses clinical challenges due to its rarity and potential for serious complications. We present a case of a 2.5 kg female neonate delivered at 37.2 weeks of gestation, diagnosed with SVT shortly after birth. Initial management included adenosine administration, which was initially ineffective until a second dose successfully reduced the heart rate. Subsequent episodes required repeated adenosine and the initiation of propranolol therapy. The neonate showed improvement with cessation of SVT episodes, weaning off respiratory support, and successful breastfeeding initiation. Follow-up at one month revealed no recurrent SVT, affirming effective management and favorable outcomes in neonatal SVT cases.

## Introduction

Arrhythmias in the neonatal period are not uncommon and may occur in neonates with a normal heart or in those with structural heart disease. Neonatal arrhythmias are classified as either benign or nonbenign. Benign arrhythmias include sinus arrhythmia, premature atrial contraction (PAC), premature ventricular contraction (PVC), and junctional rhythm; these arrhythmias have no clinical significance and do not need therapy. Supraventricular tachycardia (SVT), ventricular tachycardia (VT), atrioventricular (AV) conduction abnormalities, and genetic arrhythmias such as congenital long-QT syndrome (LQTS) are classified as nonbenign arrhythmias [1-3]. The incidence of neonatal arrhythmia is reported to be 1%-5% in all neonates [1,2].

SVT is the most common arrhythmia in children, occurring in approximately 1 in 250 neonates and 1 in 10 infants with congenital heart disease (CHD) [4]. The incidence and prevalence are reported as 13 per 100,000 and 2.25 per 100,000, respectively. In 90% of cases, re-entrant tachycardia is the underlying cause in an otherwise healthy newborn, with heart rates typically exceeding 220 beats per minute. Infants may appear more irritable and fatigued than usual. SVT is characterized primarily by tachycardia, sometimes accompanied by symptoms such as heart failure, hypotension, shock, pallor, or altered consciousness [5].

A 12-lead ECG with rhythm strips is needed to diagnose SVT. Prenatal detection of SVT is occasionally possible. Treatment is typically reserved for cases of prolonged or frequently recurring SVT episodes. In newborns, SVT often resolves spontaneously, and medications may be discontinued within 6-12 months in uncomplicated cases [6].

We report a rare case of fetal SVT detected at birth, managed with adenosine injection and subsequent monitoring in the neonatal intensive care unit (NICU).

## Case presentation

A 27-year-old woman, gravida 2, para 1, with a history of 37.2 weeks of gestation, presented with intermittent abdominal pain lasting a week. She denied any per-vaginal bleeding, leak, or discharge and had no significant medical history, particularly of cardiac or lung disorders. She was hemodynamically stable upon examination, with normal findings on the electrocardiogram (ECG) and 2D echocardiography. However, the fetal heart rate (FHR) was noted at 205 beats per minute, raising suspicion of fetal SVT.

**Figure 1:**
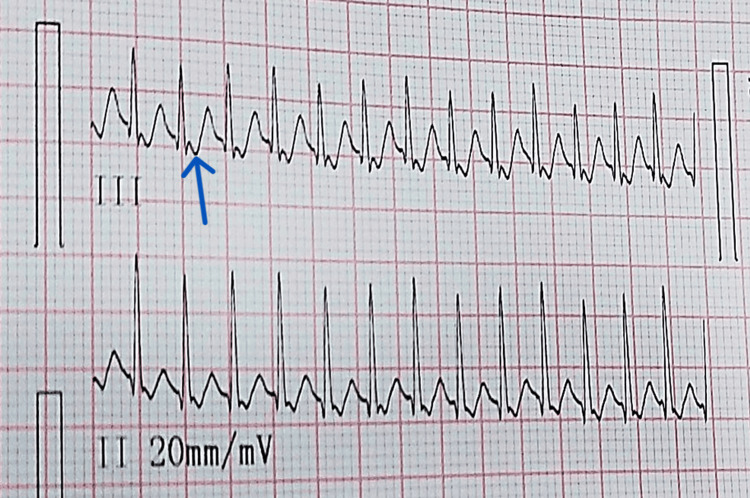
ECG (lead III) showing supraventricular tachycardia The retrograde P wave is marked by a blue arrow ECG: electrocardiogram

A cesarean section was planned, resulting in the delivery of a female infant weighing 2.5 kg with Apgar scores of 7/10 and 9/10 at one and five minutes post-birth, respectively. The baby cried spontaneously and had a heart rate of 220/minute and a respiratory rate of 52/minute, showing mild subcostal retractions indicating respiratory distress, leading to NICU admission for oxygen support via face mask and further management. In the NICU, the baby's heart rate increased to 243/minute, with a respiratory rate of 44/minute, and she maintained oxygen saturation of 100% on oxygen support. Subsequently, the baby was transitioned to indigenous continuous positive airway pressure (CPAP) and started on intravenous fluids, antibiotics, and calcium gluconate. An immediate ECG showed a heart rate of 280/minute, with a regular, narrow complex rhythm, retrograde P wave, and pseudo-S wave in the lead III, confirming SVT (Figure 1). The results of the 2D echocardiogram were within the usual range.

**Figure 2:**
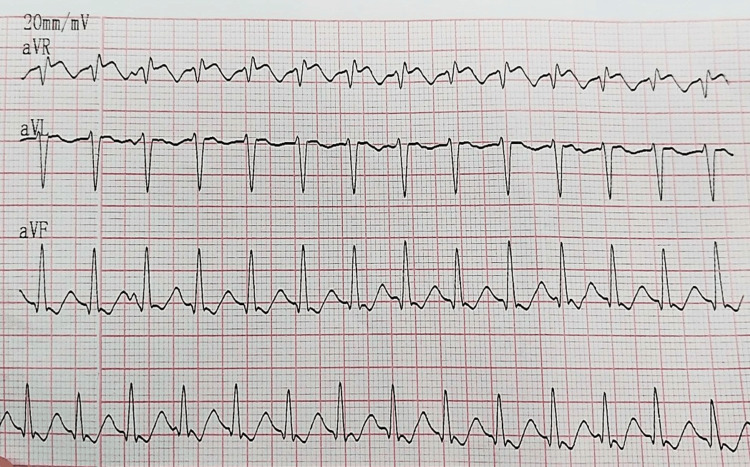
ECG after administering adenosine on the day of admission ECG: electrocardiogram

Adenosine injection was initially administered using a double-syringe technique at a dose of 0.1 mg/kg, but no effect was observed. Subsequently, a repeat dose of adenosine (0.2 mg/kg) was administered after two minutes, resulting in a reduction of the heart rate from 280 beats/minute to 178 beats/minute (Figure 2).

**Figure 3:**
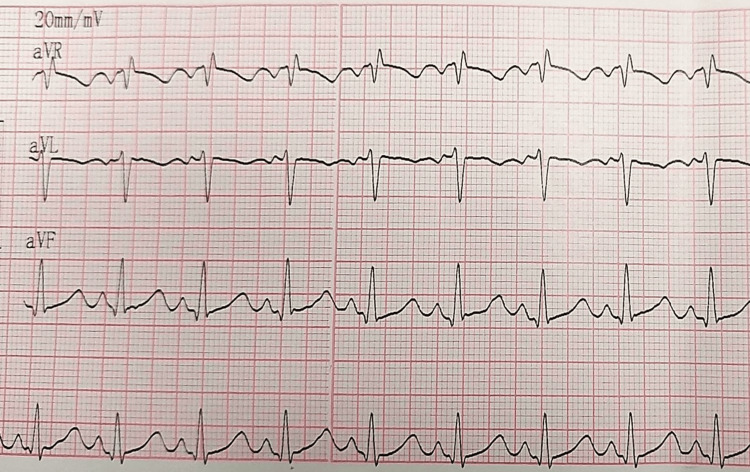
ECG confirming that the SVT settled after the administration of adenosine on day two of admission ECG: electrocardiogram

After 12 hours of admission, the baby experienced another episode of SVT with a heart rate exceeding 250 beats/minute, which was successfully terminated using an injection of adenosine. A cardiologist was consulted, and the baby was initiated on propranolol at a dose of 0.5 mg/kg every eight hours. On the second day of admission, the baby had a similar SVT paroxysm that resolved with a repeat dose of adenosine. Vitals were closely monitored in the NICU, and a subsequent ECG showed no abnormalities (Figure 3).

Subsequently, the baby did not experience any further episodes of SVT, and oxygen support was gradually discontinued. Feeding via an orogastric tube (OGT) was initiated and later transitioned to breastfeeding. The baby's condition improved, and they were discharged on day six of life while continuing oral propranolol. During the one-month follow-up, the infant exhibited adequate weight gain, was breastfeeding well, and no recurrence of SVT was observed. A 2D echocardiography performed during follow-up showed normal findings.

## Discussion

Arrhythmias, affecting 0.1%-2% of pregnant women, are among the most common cardiac manifestations during pregnancy. Some women may present for the first time with these arrhythmias during pregnancy due to the physiological stress it places on the body or because they are asymptomatic and previously undiagnosed. SVTs are the most frequent type of sustained arrhythmia observed during pregnancy [7]. Fetal tachyarrhythmia was first described by Hyman using phonocardiography and is characterized by an FHR of more than 200 bpm in a 1:1 atrioventricular ratio, known as fetal SVT. Its prevalence ranges from 1 in 1,000 to 1 in 25,000 pregnancies, accounting for 60%-80% of fetal tachyarrhythmias [8].

Fetal SVT can present in various clinical forms. It may persist with high-output cardiac failure leading to hydrops fetalis, or it may be intermittent without hemodynamic consequences. The fetus's age (younger fetuses being more vulnerable) and the duration of SVT influence the likelihood of developing hydrops fetalis. SVT can be detected as early as 13 weeks of gestation [5]. The age and duration of SVT impact its clinical presentation. Heart rates typically range from 160 to 280 beats per minute in older children and from 220 to 320 beats per minute in neonates. Symptoms such as poor feeding, pallor, vomiting, cyanosis, and irritability are often nonspecific. Ignoring these symptoms for several hours or days may lead to manifestations of heart failure [4].

Adenosine triphosphate breaks down into adenosine, a naturally occurring purine nucleoside. It acts by blocking atrioventricular nodal conduction through cardiac tissue A1 receptors, mediated by secondary messengers. These messengers affect Gi proteins, leading to decreased cyclic adenosine monophosphate levels. This process results in cardiac myocyte hyperpolarization, stimulation of potassium channels, and inhibition of L-type calcium channels, ultimately restoring normal sinus rhythm (NSR) [9].

For accurate diagnosis and treatment of symptomatic neonates, recording a cardiac rhythm strip (24-hour ambulatory monitoring, event recorders, and ECGs) is crucial. Event recorders are used frequently when symptoms occur intermittently, typically more than once a month. ECG findings often include pseudo-R waves in V1-2 and narrow complex tachycardia without discernible P waves. Holter monitoring is often impractical due to the intermittent nature of SVT symptoms in most children [4].

SVT can lead to significant morbidity, including debilitating symptoms and hospitalizations. Radiofrequency ablation (RFA) has revolutionized SVT treatment, with high cure rates exceeding 95% for atrioventricular reciprocating tachycardia and atrioventricular nodal re-entry tachycardia (AVNRT) and over 70% for atrial tachycardia [10]. Adenosine, characterized by a rapid onset and a short half-life of 5-10 seconds, facilitates quick cardioversion with minimal side effects. To ensure adequate myocardial delivery, adenosine is administered as a rapid intravenous (IV) bolus of 0.1 mg/kg (maximum 6 mg), followed by a saline flush. A second dose of 0.2 mg/kg (maximum 12 mg) can be given if needed, with a third dose possible if necessary, administered after one to two minutes [9].

## Conclusions

The case report presents a rare occurrence of SVT in a fetus detected at delivery, which was managed with an injection of adenosine and NICU monitoring. The use of adenosine was effective in reducing the heart rate and stabilizing the condition of the newborn, leading to successful treatment and early discharge. SVT is one of the most common conditions requiring emergency cardiac care in neonates. Atrioventricular re-entry tachycardia (AVRT) utilizing an atrioventricular bypass tract is the most common form of SVT in neonates. In most cases, SVT resolves spontaneously in infancy. Initial therapy for neonates typically involves pharmacological agents, with catheter ablation being a potential consideration in rare cases in which multiple drug therapies, either alone or combined, are ineffective after a thorough risk assessment. This case highlights the importance of prompt diagnosis and appropriate intervention in managing fetal SVT.
